# Impact of Helium Ion Implantation Dose and Annealing on Dense Near-Surface Layers of NV Centers

**DOI:** 10.3390/nano12132234

**Published:** 2022-06-29

**Authors:** Andris Berzins, Hugo Grube, Einars Sprugis, Guntars Vaivars, Ilja Fescenko

**Affiliations:** 1 Laser Center, University of Latvia, LV-1004 Riga, Latvia; grube.hugo@gmail.com (H.G.); iliafes@gmail.com (I.F.); 2 Institute of Solid State Physics, University of Latvia, LV-1063 Riga, Latvia; einars.sprugis@lu.lv (E.S.); guntars.vaivars@lu.lv (G.V.)

**Keywords:** nitrogen-vacancy centers, He ion implantation, diamond annealing, dense NV layers

## Abstract

The implantation of diamonds with helium ions has become a common method to create hundreds-nanometers-thick near-surface layers of NV centers for high-sensitivity sensing and imaging applications; however, optimal implantation dose and annealing temperature are still a matter of discussion. In this study, we irradiated HPHT diamonds with an initial nitrogen concentration of 100 ppm using different implantation doses of helium ions to create 200-nm thick NV layers. We compare a previously considered optimal implantation dose of ∼$10^{12}$ He${}^{+}/$cm${}^{2}$ to double and triple doses by measuring fluorescence intensity, contrast, and linewidth of magnetic resonances, as well as longitudinal and transversal relaxation times $T_{1}$ and $T_{2}$. From these direct measurements, we also estimate concentrations of P1 and NV centers. In addition, we compare the three diamond samples that underwent three consequent annealing steps to quantify the impact of processing at 1100 ${}^{{}^{\circ}}$C, which follows initial annealing at 800 ${}^{{}^{\circ}}$C. By tripling the implantation dose, we have increased the magnetic sensitivity of our sensors by 28±5%. By projecting our results to higher implantation doses, we demonstrate that it is possible to achieve a further improvement of up to 70%. At the same time, additional annealing steps at 1100 ${}^{{}^{\circ}}$C improve the sensitivity only by 6.6 ± 2.7%.

## 1. Introduction

The nitrogen-vacancy (NV) centers in diamonds are point defects consisting of a vacancy in the diamond lattice adjacent to a substitutional nitrogen atom [1]. Negatively charged NV${}^{-}$ centers, which acquire an additional electron from other substitutional nitrogen atoms, possess long coherence times of their electron and nuclear spins and can be initialized and read optically [2]. These properties made them widely studied as potential qubits and quantum sensors. Intensive studies of NV centers in the last decade have led to a large variety of sensing applications [3,4,5,6,7], which benefit from nanometer resolution and room-temperature operation of the NV-based devices, as well as from low toxicity and mechanical or chemical durability of their diamond matrix. Mainly, these applications exploit the high sensitivity of NV centers to magnetic fields via ground state Zeeman effect by using optically detected magnetic resonance (ODMR) detection [2,8,9,10].

There are several methods to create NV centers in the diamond. Nitrogen ion implantation is used in crystals with low initial nitrogen concentration [11,12,13,14,15], and the advantage of this method is the control of nitrogen distribution within the diamond; the disadvantage is the relatively high damage to the crystal during the implantation, thus introducing undesirable defects and impurities that might create charge traps, paramagnetic centers, and vacancy chains, leading to increased spectral diffusion and degraded spin coherence properties [16,17,18]. In addition, this method suffers from electron donor deficit leading to lower NV${}^{0}$ to NV${}^{-}$ charge-state conversion efficiency [19], since it is usually applied to diamonds with a low initial concentration of nitrogen. Another widely used method is electron irradiation [20,21,22,23], which creates vacancies in crystals with already sufficient nitrogen concentration. Such electron irradiation produces a minimum of undesirable defects, but the large electron energies required to create vacancies limit the control of the depth; therefore, this method is good for fabricating sensors with uniform NV distribution, where the sensing volume matches the volume of the bulk diamond. In addition, laser writing [24,25], where impulse lasers are used to create the vacancies, is not convenient for the creation of NV layers over a wide area due to limited optical depth resolution and spatial inhomogeneity as well as due to relatively high optical power required per unit area. This makes it more suited for the creation of single NVs or micrometer-sized vacancy regions.

In many applications, it is desirable to keep high spatial resolution by creating well localized NV ensembles [22,24,26,27], for example, thin NV layers for magnetic imaging [8,28,29]. In addition, dense NV ensembles are desirable since the sensitivity scales with the square root of the number of NV${}^{-}$ centers. To address these needs, helium ion implantation [30,31] has been developed in the last decade. Irradiation with lightweight helium ions creates less damage in the crystal lattice and, at the same time, gives good control over the implantation depth of several hundreds of nanometers. Such a depth is optimal for widefield magnetic imaging when an image plane is next to the sensor since the method’s spatial resolution is close to the NV layer thickness. Using more lightweight particles, such as protons [21], leads to the resolution of tens of micrometers, but using more heavy particles, such as nitrogen ions, leads to the resolutions of tens of nanometers. Thus, both methods lead to a resolution that does not match the optical resolution; therefore, these are less suited for widefield magnetic imaging. Moreover, this method allows us to create high-quality imaging sensors from inexpensive synthetic diamonds with a high concentration of nitrogen impurities. Diamonds with 100 ppm of nitrogen could potentially lead to high NV${}^{-}$ concentration if irradiated with high doses of helium ions; however, we expect some NV${}^{-}$ saturation limit primarily due to a deficit of electrons (low NV${}^{-}$/NV${}^{0}$ ratio) because of a lack of electron donors and competition from other electron acceptors. Such saturation at irradiation doses of $10^{14}$ He${}^{+}/$cm${}^{2}$ is reported in Ref. [30], but no other systematic studies of helium implantation doses for HPHT diamonds have been reported since then. The NV${}^{-}$ saturation, even at lower irradiation doses, is reported in nanometer-thick profiles of NV centers of CVD diamonds [32]. The recent studies of NV imaging [28,33] conservatively used $10^{12}$ He${}^{+}/$cm${}^{2}$ irradiation doses, which might be sub-optimal for HPHT diamond applications.

All aforementioned irradiation methods require annealing to promote the migration of vacancies to substitutional nitrogen defects and heal the crystal; however, the optimal annealing conditions are still a cause for the debate. For example, there is some uncertainty related to the effects that the longer annealing times and higher temperatures bring: on the one hand, such treatment reduces the concentration of radiation-induced defects while maximizing the NV${}^{-}$/NV${}^{0}$ ratio in nitrogen ion-implanted samples and increasing the $T_{2}$ relaxation time [12], but on the other hand, in such samples, the higher annealing temperatures leads to a rise in the concentration of the H3 center (an emission center formed by a vacancy together with two nitrogen atoms (NVN)) [34] that might lead to adverse effects on P1 to NV${}^{-}$ conversion efficiency. In general, existing experimental studies of annealing are hardly comparable, as they are performed using different NV preparation methods and diamonds. At the same time, very different annealing procedures are reported in the case studies. There is a body of publications using annealing in temperature interval 750 ${}^{{}^{\circ}}$C to 900 ${}^{{}^{\circ}}$C and annealing times from 1 to 2 hours [30,31,35,36] under vacuum or Ar and H${}_{2}$ mixture. Some research studies apply longer annealing times [37,38] and higher temperatures [12,32,34,39] or both [18,22,28]. It is likely that in many cases the temperature range 750 ${}^{{}^{\circ}}$C to 900 ${}^{{}^{\circ}}$C is defined by maximum temperature achievable by majority of conventional ovens. Moreover, additional annealing in air at temperatures around 500 ${}^{{}^{\circ}}$C is sometimes used to improve luminescence of NV centers [35,40], but such a treatment is off topic for our study.

In this research, we set out to find the trends of fluorescence intensities, contrast, and FWHM of ODMRs, as well as $T_{1}$ and $T_{2}$ relaxation times for three HPHT diamond samples with a nitrogen concentration of ∼100 ppm, which we irradiated with standard (previously used [28,33]), double and triple ${}^{4}$He${}^{+}$ doses, to create ∼200 nm thick NV layers. We hypothesized that doubling or tripling the He${}^{+}$ implantation dose of an HPHT diamond would proportionally increase the concentration of NV${}^{-}$ centers and, therefore, could lead to the fabrication of imaging sensors with higher magnetic sensitivity. We also investigate changes in these parameters after applying each of the three consecutive annealing steps: first at maximum temperatures of 800 ${}^{{}^{\circ}}$C and two subsequent annealing steps at a maximum temperature of 1100 ${}^{{}^{\circ}}$C.

## 2. Materials and Methods

### 2.1. Fabrication

**Figure 1:**
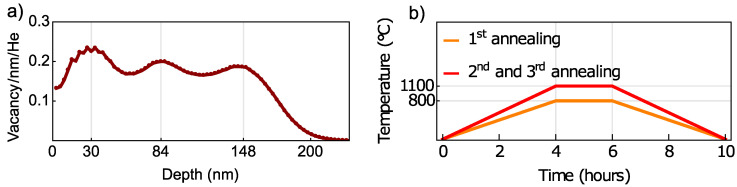
**Fabrication of samples:** (**a**) SRIM vacancy-depth profile for fabrication of 200-nm-thick NV layer close to the diamond surface. (**b**) Time–temperature graphs for the three annealing steps.

In measurements, we used three HPHT type Ib diamond crystals (Sumitomo Electric) with a (110) surface polish and dimensions of 2 mm × 2 mm × 0.06 mm. All three crystals (samples F1, F2, and F3) were initially cut from one 0.5 mm thick crystal by Almax easyLab BVBA. We performed Stopping Range of Ions in Matter or SRIM simulations [41] to determine the implantation parameters required for fabrication of 200-nm-thick NV layer close to the diamond surface (Figure 1a).

The three crystals were irradiated with He ions at three energies: 33 keV, 15 keV, and 5 keV, with doses represented in Table 1 by Ion Beam Services SA. After the implantation, the crystals underwent three steps of annealing with 6 h boiling at 200 ${}^{{}^{\circ}}$C in triacid (1:1:1 mixture of nitric: perchloric: sulfuric acids) before and after each step. The first annealing was performed at 800 ${}^{{}^{\circ}}$C for two hours, and the last two annealing steps were performed at 1100 ${}^{{}^{\circ}}$C (Figure 1b). All annealing steps were performed under vacuum; in all cases, the ramp-up and cool-down times were 4 hours. The first annealing step was performed by using a Setaram LABSYS evo STA system and in 1 $\times \;10^{-2}$ ± 0.1 $\times \;10^{-2}$ mbar vacuum, but the last two annealing steps were performed in tube furnace (OTF-1200X-S from MTI corporation) in 1 $\times \;10^{-5}$ ± 0.3 $\times \;10^{-5}$ mbar vacuum (Edwards T-Station 85H Wet). After each annealing step, a complete set of measurements was performed for each sample in six equidistant spots along a diagonal of the sensor’s top surface. We take a mean value of all measurements in the six spots with its standard error as an error bar.

### 2.2. ODMR Measurements

Firstly, we characterize samples by measuring and analyzing their CW ODMR spectra [2,8]. Zeeman splitting between ground-state electronic spin levels is induced in the NV${}^{-}$ centers by a bias magnetic field applied along with one of four possible NV axes. We detect a fluorescence spectrum containing a series of separated magnetic resonances by sweeping a transverse to the NV axis microwave field. We fit the spectrum with a series of Lorentzian profiles to quantify ODMR contrast, full width at half maximum (FWHM), and fluorescence intensity off-resonance. Both contrast and FWHM were obtained from the resonance fit at spin transition |0〉↔|−1〉. Measuring the FWHM linewidths, we keep the MW power weak enough to avoid any power broadening.

The off-resonance fluorescence intensity gives information about the NV = NV${}^{-}$+ NV${}^{0}$ concentration in the samples. Other fluorescent centers that contribute to the fluorescence, such as H3 center (NVN) [34] or helium vacancies (HeV) [37,42] are much less abundant or do not radiate in the detection frequency range. The contrast (the relative fluorescence intensity difference in ODMR signal on and off-resonance) provides further information about the charge of NV centers as it is proportional to the NV${}^{-}$/(NV${}^{-}$+NV${}^{0}$) ratio. The FWHM informs about the inhomogeneity of the NV environment that represents a limiting factor for the magnetic field sensitivity of CW ODMR methods. This FWHM is directly related to inhomogeneously broadened transverse relaxation time $T_{2}^{*}$ and is caused by several NV spin ensemble dephasing sources, such as interactions with nuclear ${}^{13}$C bath spins [43,44,45], crystal-lattice strain fields over the diamond [43,46,47], and measurement-related artifacts such as magnetic field gradients over the collection volume and temperature fluctuations [43,48].

### 2.3. Relaxation Measurements

Secondly, we characterize samples by measuring and analyzing dynamics of NV ensembles by using relaxometry measurements: longitudinal (spin-lattice) relaxation time $T_{1}$ that characterizes NV spin ensemble dephasing mainly due to cross-relaxation within the strongly interacting bath of NV${}^{-}$ spins [49,50]; the transverse relaxation time $T_{2}$ that characterizes homogeneous decoherence of the prepared state of the NV ensemble, and are mainly caused by the interaction of NV${}^{-}$ with the spin bath of substitutional nitrogen atoms (P1 centers) [49,51]. For a detailed description and explanation of these relaxometry measurement sequences, see references [2,8,10].

The used microwave sequences are preceded by a 5μs long initializing laser pulse to prepare the population in the |0〉 ground state. For the $T_{1}$ sequence, we use a {(π)−τ} and for $T_{2}$ (Hahn echo) we use {π/2−τ/2−π−τ/2−π/2} MW impulse sequences, where τ is interrogation time, π denotes microwave pulse that transfers NV${}^{-}$ population between ground-state electronic spin levels, but π/2 microwave pulse creates a superposition of these levels. We start every second run of the $T_{1}$ sequence with a π pulse to alternate interrogation of the population on |0〉 and |+1〉 spin levels. The same alternation for the Hahn echo sequence was performed by shifting a phase of the last π/2 pulse relative to the first π/2 pulse by $90^{{}^{\circ}}$ in every second run. The 5μs long read-out laser pulse induces a fluorescence pulse similar to the initializing pulse at the start of the sequence but with a signal depression at the beginning. This relative amplitude of the signal is proportional to the population of the interrogated level. From the difference between the fluorescence signals of the initializing pulse and read-out pulse, we calculate a common-noise-free ODMR contrast, plotted as a function of the increasing interrogation time τ. The resulting decay plots are fitted with exponential functions in the form *C* exp${\left(-\tau /T\right)}^{p}$ where *C* is contrast, *T* is a relaxation constant, but parameter *p* is 1 for fitting longitudinal decays or 3/2 for fitting transverse decays [49].

### 2.4. Apparatus

**Figure 2:**
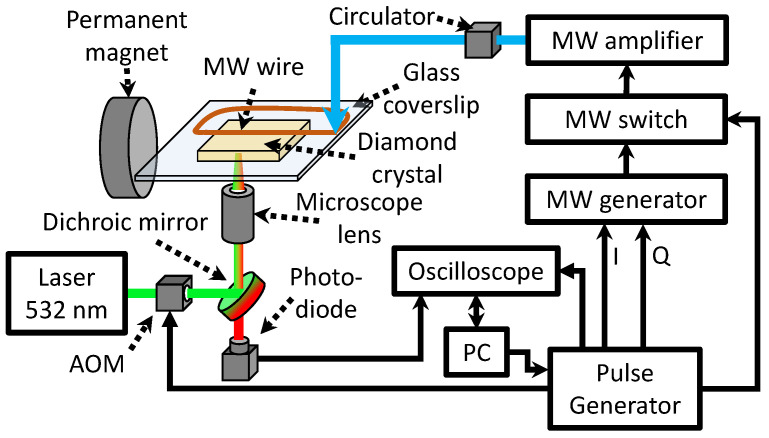
**Schematic of the experimental apparatus.** AOM: acousto-optic modulator; I and Q: phase shift control; PC: the personal computer.

The experimental setup for the characterization of samples is depicted in Figure 2. During the measurements, the diamond sample was placed on a coverslip in an epifluorescent microscope. The NV excitation and fluorescence detection were performed through the same infinity-corrected oil-immersion 100× microscope objective with a numerical aperture of 1.25 (ZEISS). The NV centers were exposed to 200 mW radiation guided by a multi-mode optical fiber and lens system from a Coherent Verdi V-18 laser. The NV fluorescence (650−−800 nm) is separated by a dichroic mirror (Thorlabs DMLP567R) and is measured on an avalanche detector (Thorlabs APD410A/M) through a long-pass filter (Thorlabs FEL0600). During the measurements, we illuminate the NV layer in a region with a diameter of 30μm.

The bias magnetic field $B_{0}\approx 6$ mT is produced by a neodymium permanent disk magnet and aligned along with one of the NV axes in the plane of the diamond plates (polished along the (110) direction). A microwave generator (SRS SG384) produces the MW field for the measurements. The microwaves subsequently pass through an amplifier (Mini-Circuits ZHL-16W-43+) and circulator and are delivered by a copper wire with a diameter of 50μm to the diamond sensor.

The relaxation measurements are controlled by a TTL pulse card (PBESR-PRO-500 by SpinCore). Microwave pulses are generated using the microwave generator in the I/Q modulation mode. The microwave amplitude and phase are controlled on a ≲10 ns timescale utilizing a series of TTL-controlled switches (Mini-Circuits ZASWA-2-50DR). Laser pulses are generated by passing the continuous-wave laser beam through an acousto-optic modulator (MT200-A0.5-VIS by AA Optoelectronic). An oscilloscope measures the avalanche detector output voltage, reporting fluorescence time traces to the computer controlling the experiment.

## 3. Results and Discussion

### 3.1. Fluorescence Intensity, Contrast, Linewidth, and $T_{2}^{*}$

**Figure 3:**
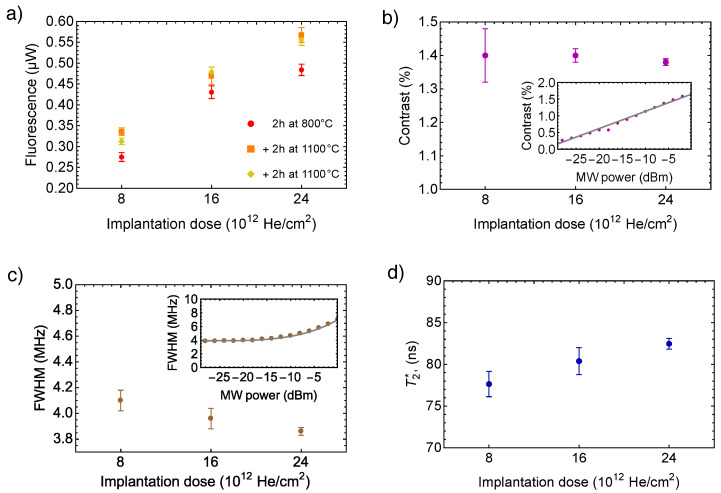
**ODMR measurements:** (**a**) Off-resonance fluorescence intensity for three subsequent annealing steps versus cumulative implantation dose. (**b**) Contrasts of the ODMR signals at |0〉↔|−1〉 spin transition versus the cumulative implantation dose. The contrasts are shown for −5 dBm of MW power (see the inset). (**c**) The FWHM of the ODMR signals at |0〉↔|−1〉 spin transition versus the cumulative implantation dose. The FWHMs linewidths are shown for −25 dBm of MW power (see the inset). (**d**) Calculated from the FWHM inhomogeneously broadened transverse relaxation $T_{2}^{*}$ versus the cumulative implantation dose. A complete breakdown of implantation doses can be found in Table 1. The error bars represent the standard error (SE) of the data. All ODMR data except the fluorescence intensity show no significant correlation with the annealing temperature or duration; therefore, the contrasts, FWHMs, and $T_{2}^{*}$ after averaging over all three annealing steps are shown for simplicity.

The results of ODMR measurements are summarized in Figure 3. As expected, the fluorescence intensity (Figure 3a) is larger when larger He${}^{+}$ implantation doses are used because higher vacancy concentration leads to a higher proportion of the P1 centers converted to NV centers. There is also a pronounced fluorescence intensity increase between the first annealing at 800 ${}^{{}^{\circ}}$C and the second annealing at 1100 ${}^{{}^{\circ}}$C, and additional third annealing at the last temperature does not lead to a notable increase in intensity anymore. That means free vacancies do not travel fast enough to create all potential NV centers during the first annealing. More prolonged annealing at the same lower temperature would likely produce the same increase in intensity. In other words, besides healing the lattice, the annealing at 1100 ${}^{{}^{\circ}}$C accomplished the started work of the previous annealing at a lower temperature by moving on free vacancy toward substitutional nitrogen atoms; however, previous research [12] shows that increasing the annealing temperature to ≈1100 ${}^{{}^{\circ}}$C enhances the $T_{2}$ relaxation time (discussed in the next section). An obvious question then arises if just one annealing at 1100 ${}^{{}^{\circ}}$C would be enough instead of the more complicated two-step annealing.

The ODMR contrast versus the implantation dose presented in Figure 3b shows no change within error bars. It also does not have a significant correlation with the annealing temperature or duration, and for simplicity, it is shown here after averaging over all three annealing steps. The contrast is proportional to the ratio NV${}^{-}/$NV${}^{0}$, as only the negative NV centers contribute to the ODMR signal, but fluorescence from the neutral NV centers contributes to the signal background alone. Such a ratio could drop when most of the P1 centers are converted to NV centers [30,32] because P1 centers are the main donors of the electrons for the negative NV centers. The higher the implantation dose, the higher concentration of vacancies, which leads to a higher proportion of the P1 centers (single nitrogen defects) converted to NV centers. As a rule of thumb, the concentration of the NV centers should not be larger than that of P1 donors because a further increase in the NV concentration would not lead to the creation of new negative NV centers. The “standard” implantation dose of $10^{12}$ He${}^{+}/$m${}^{2}$ previously used in Ref [28,33] was chosen because of an estimate that it leads to creation of ≈50 ppm of NV centers. In fact, not all population of NV centers acquires the negative charge regardless of the abundance of P1 centers, and usually, less than 30% of the ensemble of NV centers is negatively charged [39,52]. Therefore, when planning the NV fabrication, only the concentration of negatively charged NV centers $\rho_{{\mathrm{NV}}^{-}}=\rho_{\mathrm{NV}}\times \delta$, where δ is an expected NV${}^{0}$-to-NV${}^{-}$ charge-state conversion efficiency of the NV sensor, should be kept equal to the P1 center concentration. Furthermore, we likely do not see saturation in the NV${}^{-}$ concentration (drop in the contrast) even after the triple dose because we did not reach this match of P1 and NV${}^{-}$ concentrations.

The FWHM linewidth and relaxation time $T_{2}^{*}$ associated with it versus the implantation dose are presented in Figure 3c,d, correspondingly. The FWHM does not have a significant correlation with the annealing temperature or duration, and for simplicity, it is also shown after averaging over all three annealing steps. The relaxation time calculated from linewidth Γ as $T_{2}^{*}=1/\left(\pi \Gamma \right)$ [39,53] is sensitive to magnetic noise of various origin. Because of a decrease in the concentration of magnetically noisy P1 centers due to their combination with free vacancies, we expect the mitigation of the NV spin dephasing [43] and larger values of $T_{2}^{*}$ when higher implantation doses are used. Moreover, the P1 centers could be converted into H3 centers or NVN, which are not detected by the experimental setup since they radiate at 505.8 nm [34]; however, the concentration of H3 centers is by two orders less than the concentration of P1 centers [34,54], so their contribution to the dephasing is relatively small. The $T_{2}^{*}$ plot on Figure 3d qualitatively supports the dominant role of P1 centers in the dephasing of NV spins.

Values of the fluorescence intensity *I*, the contrast *C* (the relative difference in ODMR signal on/off resonance), and the FWHM linewidth Γ allow us to compare the sensitivity of the samples that is the minimum detectable magnetic field of a Lorentzian ODMR signal as [28]

(1) $$B_{\min}\propto \frac{\Gamma }{C\sqrt{I}}.$$

By normalizing the sensitivities obtained with Equation (1) to $B_{\min}$ of the sample F1 with the smallest “standard” implantation dose, we found the relative improvement of the sensitivity for the sample F2 by 22±5% and the sample F3 by 28±5%. At the same time, an average gain of the sensitivity of all samples between the first annealing at 800 ${}^{{}^{\circ}}$C and the second annealing at 1100 ${}^{{}^{\circ}}$C is a modest 6.6 ± 2.7%.

### 3.2. Longitudinal and Transverse Relaxations

**Figure 4:**
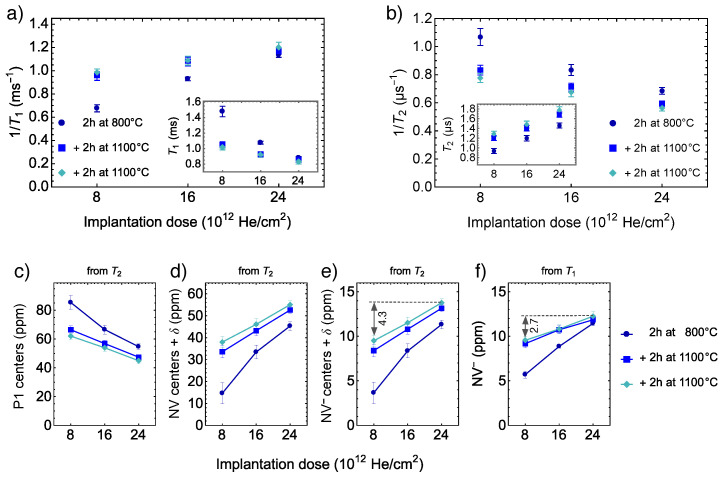
**Relaxation measurements:**(**a**) Relaxation rate 1/$T_{1}$ for three subsequent annealing steps versus cumulative implantation dose. The inset shows the same plot in $T_{1}$ units. (**b**) Relaxation rate 1/$T_{2}$ for three subsequent annealing steps versus cumulative implantation dose. The inset shows the same plot in $T_{2}$ units. **Estimates of concentrations:**(**c**) Concentration of P1 centers estimated from $T_{2}$. (**d**) Concentration of NV centers estimated from $T_{2}$. (**e**) Concentration of NV${}^{-}$ estimated from $T_{2}$. Symbol δ denotes small concentrations of other N-containing defects. (**f**) NV${}^{-}$ concentration estimated from $T_{1}$. All estimates are shown versus cumulative implantation dose for three subsequent annealing steps. The solid lines are shown to guide the eye.

Measured longitudinal relaxation rates versus cumulative implantation doses are plotted in Figure 4a. The inset shows the same plot in $T_{1}$ units. This relaxation characterizes the rate with which the spin population decays back to a thermally mixed state mainly due to cross-relaxation interactions with a bath of other NV${}^{-}$ centers [49]. The density of NV${}^{-}$ bath in our samples varies with the concentration of vacancies (implantation dose) and the completeness of the annealing procedure. As a result, we see an increase in the $1/T_{1}$ rate both due to higher implantation doses and partially due to the second annealing step. In the perspective of $T_{1}$, the effect of the second annealing is due to the shortness of the first annealing step rather than the larger annealing temperature. This effect is apparent for the sample with the largest implantation dose, which is not improved with the second annealing step. Indeed, when a large implantation dose leads to a dense network of vacancies, a vacancy needs a shorter travel time before combining with a nitrogen atom.

Figure 4b depicts measured $1/T_{2}$ rates versus cumulative implantation doses. The inset shows the same plot in $T_{2}$ units. The $1/T_{2}$ is the rate with which electron spins of the NV${}^{-}$ centers are homogeneously dephased, and it is proportional to nitrogen (P1 centers) concentration—the main source of the spin dephasing [49]. The larger the implantation dose, the larger part of the initially presented P1 centers can be converted into other kinds of defects [1]. Note that unlike for the $T_{1}$ relaxation, the $T_{2}$ of the sample with the largest implantation dose is increased after the second annealing, which may be a result of P1 conversion into H3 centers [34,54]. It may also be a result of a drop in concentration of possibly present vacancy chains, as at ≈1100 ${}^{{}^{\circ}}$C their concentration is greatly reduced, effectively reducing the concentration of vacancy-related paramagnetic defects [12]. In both cases, from the perspective of $T_{2}$ time, the annealing at temperature 1100 ${}^{{}^{\circ}}$C is favorable. In our case, the three samples initially had the same nitrogen concentrations; by introducing vacancies and creating NV centers, we effectively decreased the P1 concentration and increased the $T_{2}$ time. The sensitivity of pulse magnetometry methods is usually limited by the $T_{2}$ time [2], which in turn may be limited by the $T_{1}$ time, as $T_{2}^{max}\approx 0.5T_{1}$ [55]. The observed drop in the $T_{1}$ time does not affect the potential magnetic sensitivity because, for our samples, $T_{1}$ times is three orders of magnitude larger than $T_{2}$ times.

### 3.3. Estimates of Concentrations

We go further by using the previously published dependency of $T_{2}$ on the concentration of P1 centers [49] to estimate the concentration of P1 centers indirectly. We use an equation $1/T_{2}=x/T_{\mathrm{NV}-P1}$, where $T_{\mathrm{NV}-P1}$ is the P1-dominated NV decoherence time per unit concentration *x*. From the fit of the numerical simulation data, we extract $T_{\mathrm{NV}-P1}$ = 80 μs ppm. Note that we do not use the experimental dependence from the same Ref. [49] since it leads to concentrations of P1 centers that are much larger than a known initial concentration of nitrogen, which the manufacturer gives as ≈ 100 ppm. Concentrations of P1 centers estimated from the measured $T_{2}$ relaxation times are depicted in Figure 4c. These estimates are used further to determine the total concentration of NV ≈ NV${}^{-}$ + NV${}^{0}$ by subtracting the P1 concentration obtained from $T_{2}$ from the known initial nitrogen concentration, see Figure 4d. Then, by using a conservative value of the NV${}^{0}$ to NV${}^{-}$ charge-state conversion efficiency of 25%, we estimate the NV${}^{-}$ concentration, see Figure 4e. Based on the contrast measurement (Figure 3b), we assume that the conversion efficiency is the same for all three samples. We also assume that P1 centers may be converted into a tiny but not negligible concentration of nitrogen-containing defects (denoted as δ) other than NV${}^{0}$ and NV${}^{-}$ centers.

Similarly, we use a measured dependency of $T_{1}$ on the concentration of NV${}^{-}$ centers, which is published in Ref. [23]. From the fit of the experimental data we found a linear equation $1/T_{1}=1/T_{1,\mathrm{other}}+x/T_{\mathrm{NV}-\mathrm{NV}}$, where $T_{\mathrm{NV}-\mathrm{NV}}=0.08$ ms·ppm is the dipole–dipole interactions driving relaxation time per unit concentration *x* and the relaxation time $T_{1,\mathrm{other}}=4.45$ ms accounts for other decoherence mechanisms. Concentrations of NV${}^{-}$ centers estimated from the measured $T_{1}$ relaxation times are depicted on Figure 4f. This estimate leads to values about 10 ppm, similar to values estimated from $T_{2}$ relaxation, supporting our assumption about the charge-state conversion efficiency of 25%.

However, the slopes of the dependencies on the implantation dose are different. After the third annealing step, the estimates of NV${}^{-}$ concentration derived from the $T_{2}$ have an increment by 4.3 ppm between the minimum and the maximum implantation doses, but the estimates derived from the $T_{1}$ have an increment only by 2.7 ppm between the same doses, see Figure 4e,f. The difference of the increments (slopes) has a physical meaning, and this could indicate one of the two (or a combination of): a small concentration of N-containing defects δ, which are neither P1 neither NV centers or a decrease in paramagnetic defects that are not related to nitrogen. Following the first hypothesis, the slopes after the first annealing at 800 ${}^{{}^{\circ}}$C show a zero difference δ within error bars. This points to the H3 centers [34,54], whose formation is intensified at larger annealing temperatures. The previously reported [34,54] concentration of H3 defects after an annealing at 1150 ${}^{{}^{\circ}}$C is ≈1% of the NV center concentration, that is 0.6 ppm for 55 ppm of NV centers; therefore, this expected concentration of H3 defects is of the same magnitude as the concentration δ=1.5±0.7 ppm derived from the difference of the estimates in Figure 4e,f.

Following the second hypothesis, of a decrease in paramagnetic defects that are not related to nitrogen, one can argue that by increasing the annealing temperature to ≈1100 ${}^{{}^{\circ}}$C, the concentration of vacancy chains drops dramatically, effectively reducing the concentration of vacancy related paramagnetic defects [12]. This would enhance the $T_{2}$ relaxation time, but it would not change the $T_{1}$ relaxation time, as the $T_{1}$ time is sensitive only to the changes NV${}^{-}$ bath. As a result, the NV${}^{-}$ concentration estimations (Figure 4e,f) from the $T_{1}$ and $T_{2}$ could be shifted because of the defects not related to nitrogen.

## 4. Summary and Outlook

With this research, we set out to find an optimal implantation dose and annealing parameters to maximize the sensitivity of an NV-based sensor. Our efforts were focused on relatively cheap HPHT diamonds with high initial nitrogen concentration (100 ppm), as this kind of diamond-based sensors would be of interest for mass-production of high sensitivity sensors. Our measurements clearly show that striving for a higher sensitivity sensor (higher NV${}^{-}$ concentration) does not necessarily lead to degrading its properties.

**Figure 5:**
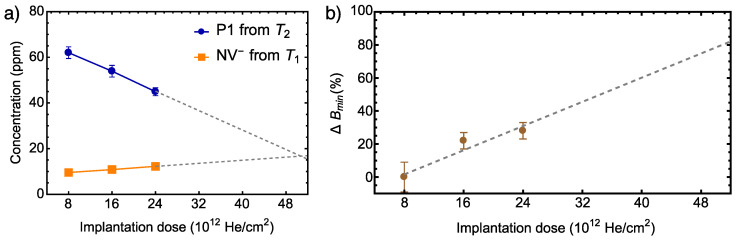
**Projected values:** (**a**) Linear extrapolations of P1 and NV${}^{-}$ concentrations to higher implantation doses. The optimal dose is expected at $0.5\times 10^{14}$ He${}^{+}$/cm${}^{2}$ where the P1 concentration is equal to the NV${}^{-}$ concentration. (**b**) The linear extrapolation to higher implantation doses of relative improvement of sensitivity (minimum detectable magnetic field $B_{\min}$, see Equation (1)).

Since our data show a linear increase in NV${}^{-}$ concentration upon increasing the He${}^{+}$ implantation dose, we can conclude that from the sensitivity perspective, it is more lucrative to use thrice the ion implantation doses than reported previously. Assuming that at least one P1 center is needed as an electron donor for each NV${}^{-}$, we estimate the maximum cumulative dose that could be used to saturate the NV${}^{-}$ concentration for the ≈ 200 nm thick layer with an initial nitrogen concentration of 100 ppm. For this, we fit our data with linear functions and extrapolate to a dose, where the P1 center concentration is equal to the NV${}^{-}$ center concentration. The maximum cumulative dose obtained in this way is ≈0.5× 10${}^{14}$ He${}^{+}$/cm${}^{2}$ (see Figure 5a). This is also consistent with the estimations of He${}^{+}$ dose and NV${}^{-}$ concentrations for similar samples in Ref. [30], which reports a sign of saturation at such a dose.

Similarly, we estimate a relative improvement of sensitivity for the dose of $0.5\;\times \;10^{14}$ He${}^{+}$/cm${}^{2}$ by interpolating the values of relative obtained from Equation (1), see Figure 5b. If we optimistically assume a linear growth of the sensitivity, then we could expect a significant potential improvement up to 70%. Half of this improvement is already achieved in this study.

Dependencies of different measured characteristics on annealing suggest that the annealing only at 800 ${}^{{}^{\circ}}$C does not deliver the optimal charge-state conversion efficiency. Only after the additional 2 h annealing at 1100 ${}^{{}^{\circ}}$C the fluorescence reaches its maximum, and relaxation time $T_{2}$ reaches its extreme value. These characteristics might be connected to the reduction in the vacancy chain-related paramagnetic defects observed at temperatures above 1100 ${}^{{}^{\circ}}$C [12], or it might be connected to the conversion of P1 centers into H3 centers [34] that also increases the $T_{2}$ time. Our results show that the average relative improvement of sensitivity between the first annealing at 800 ${}^{{}^{\circ}}$C and the second annealing at 1100 ${}^{{}^{\circ}}$C is 6.6 ± 2.7%. While we do not see perspectives for further improvement of the sensitivity by adjusting the annealing procedure, we conclude that the annealing at 1100 ${}^{{}^{\circ}}$C should not be neglected during the fabrication of NV sensors.

## Figures and Tables

**Table 1:** He ion implantation doses and energies used for fabrication of samples F1, F2, and F3.

Energy, keV	Normalized Dose	Dose ($10^{12}$ He${}^{+}$/cm${}^{2}$)		
		F1	F2	F3
33	1.0	4.0	8.0	12.0
15	0.5	2.0	4.0	6.0
5	0.5	2.0	4.0	6.0
	Total:	8	16	24

## Data Availability

The data presented in this study are available on request from the corresponding author.

## Abbreviations

The following abbreviations are used in this manuscript:

NV	nitrogen-vacancy center
P1	substitutional nitrogen atoms in diamond crystal
NVN	nitrogen-vacancy-nitrogen center (H3 center)
HeV	helium vacancies
ODMR	optically detected magnetic resonance
FWHM	full width half maximum
CVD	chemical vapor deposition
HTHP	high temperature high pressure
SE	standard error
